# Factors Influencing Men Entering the Nursing Profession, and Understanding the Challenges Faced by Them: Iranian and Developed Countries’ Perspectives

**DOI:** 10.5812/nms.12583

**Published:** 2013-12-09

**Authors:** Vahid Zamanzadeh, Leila Valizadeh, Reza Negarandeh, Morteza Monadi, Arman Azadi

**Affiliations:** 1 Department of Nursing, Tabriz University of Medical Sciences, Tabriz, IR Iran; 2 Department of Nursing, Nursing and Midwifery Care Research Center, Tehran University of Medical Sciences, Tehran, IR Iran; 3 Faculty of Education and psychology, Alzahra University, Tehran, IR Iran; 4 Department of Nursing, Ilam University of Medical Sciences, Ilam, IR Iran

**Keywords:** Nurses, Male, Gender Identity, Nursing, Iran, Review

## Abstract

**Context:**

Men entering the nursing profession have been investigated from several different perspectives. Due to male gender characteristics and existing public image, nursing is often not considered as a career choice by men. Whether nursing would benefit from increased number of men is a key question in the literature. The purpose of this integrative review of the literature was to identify factors influencing men to enter the nursing profession. In addition, it sought to understand the challenges they are confronted within this profession.

**Evidence Acquisition:**

A systematic search of the existing literature was performed using an Internet search with broad keywords to access related articles in both Persian and English databases. Finally, 34 studies (written between 2000 and early 2013) were selected and surveyed.

**Results:**

Most of the studies were conducted in developed counties. The review identified reasons why males choose nursing, and other challenges facing men entering and working in nursing. Themes that emerged from the literature include educational and societal barriers experienced by men in nursing, recruitment, career choice, and role strain.

**Conclusions:**

Regarding men’s influences on professional development, and also the importance of gender-based caring, policies for recruitment and retention of men in nursing must be followed hastily. However, there is a need for further research regarding the challenges faced by men entering nursing, in both Iran and other developing countries.

## 1. Context

Although the number of men entering nursing profession is on the rise, there has not been a significant increase in the percentage of men in nursing (1). In Canada and the United States, only 5% of nurses are men (1, 2). In England and Ireland men also represent 10% and 4% of registered nurses (RNs), respectively (3, 4). In Iran, in the final years of war, from 1985 to 1988, about 50 percent of the baccalaureate students admitted to the nursing program were men; however, after the war, it decreased to approximately 20 percent (5). According to Khosravi et al. in 2006 about 23% of nurses in Iran were men (6). Although the number of men entering the nursing profession is on the rise, the gender imbalance continues to exist for men in nursing as more male than female nurses are leaving the profession (7, 8). Hooshmand Behabadi et al. reported that poor image of nursing and ambiguous social status of nurses were major causes of leaving nursing by Iranian male nurses (9). A significant proportion of nursing literatures written by male nurses have paid attention to their dilemma in a women dominated career (3, 4, 10, 11). Men difficulties in female dominant occupations has been cited as “ignorance outside the profession and prejudice inside it” (11, 12). Feeling of isolation, experiencing enmity from female colleagues, and having to understate masculinity are examples offered by male nurses (13). Despite the fact that men appear to be marginal because of being a minority in the profession, this has also became an advantage for them, because most of prestigious positions are occupied by them (14). O’Lynn stated that because of universal nursing shortage, the profession should no longer focus on an unlimited supply of women to become nurses (11). Nursing recruitment from men can help supply the profession’s current and future vacancies (4). Nursing recruitment of men has two main advantages; first, it alleviate nursing shortage through introducing nursing to half of population who perceive nursing as a feminine profession, and entering men into nursing can cause development of professional value within other health professions (7). Although the number of men entering nursing schools has increased after the Islamic revolution in Iran, nursing is still seen as a feminine profession which exemplifies caring and altruism allied with the traditional female role (15). Although Iranian patients prefer to be cared for by nurses of the same sex, many of them are astonished when they are cared for by men nurses (16). According to Nasrabadi et al. many Iranian nursing students, particularly male nursing students, aspire to get a university degree, while they do not wish to become a nurse or get a nursing degree. Thus, there is considerable job dissatisfaction among Iranian male nurses; hence, a high level of turnover (5). Lack of consensus among the literature regarding impact of men in and on the profession is evident. In fact one of the main concerns in the literature regarding the increasing number of men in nursing is related to potential threats that men can pose to their female counterparts’ autonomy. On the other hand, there has been concerns about the low number of men in nursing and attention has been paid to development of strategies to attract more men into the profession (11, 13, 14). Therefore, the purposes of this review paper was to discuss the current state of knowledge in the area of factors influencing men to enter nursing, and challenges faced by them in this female predominant profession through the use of an integrative review of the literature.

## 2. Evidence Acquisition

A search was conducted via PubMed (NLM), CINAHL, ProQuest, and Web of Science databases. Search terms included ‘Men OR Male AND Nursing’, ‘Men in Nursing Education’ and, ‘Gender and Nursing’. These keywords equivalents in Farsi were searched in Persian databases such as SID and Magiran. By using these broad terms, the search yielded more than 500 results, though not all of them were relevant to the topic under study. Next, the terms were narrowed down several times until we found articles reflecting the terms and literature specific to our topic. Finally, 34 studies were selected for review, most of them being international. A few studies conducted in Iran and published in national and international journals were also included in this review. The articles selected for review consisted of primary studies, which explored issues and trends relevant to men experiences in nursing education, and practices which were performed using quantitative and particularly qualitative methods (Table 1). Most studies used to inform this topic were written from 2000, until early 2013. Most written about our topic were around these themes: educational and societal barriers experienced by men in nursing (1, 4, 11, 13, 16- 19), recruitment (5, 7, 8, 15, 20- 22), career choice (2, 10, 23- 29), and role strain (3, 12, 30- 37). This review aimed to answer some questions about men nurses including:

1. their characteristics,
2. major motivations influencing them to become nurses,
3. main challenges faced by male students and nurses within the profession,
4. and whether they earn some advantages in a female predominant career.

**Table 1.** Summary of Main Research Included in This Review

Authors	Type of study	Sample	Context	Findings
**O’Lynn (2004) (11)**	Survey	Male nurses (n = 200)	The USA	Finding suggested that the barriers men face in nursing school are pervasive, consistent, and have changed little over time.
**Ellis et al. (2006) (17)**	Descriptive qualitative	Male nurses (n = 13)	The USA	The findings showed a lack of satisfaction of nursing school, the importance of career opportunities, and the need for more male nurse educators.
**Stott (2007) (13)**	Thematic analysis	Male nursing students (n = 8)	Australia	Findings emphasized the fact that male nursing students tended to feel isolated and excluded from an academic and clinical perspective.
**Meadus & Twomey (2011) (1)**	Phenomenology	Male nursing students (n = 27)	Canada	The experiences of the students revealed issues related to gender prejudice in nursing education, practice, and societal perceptions, where it is believed that nursing is not an appropriate career choice for men.
**Keogh & O'Lynn (2007) (4)**	Survey	Male nurses (n = 100)	Ireland	The findings showed some negative experiences that many male students encountered in nursing schools.
**Wang et al. (2011) (18)**	Phenomenology	Male nurses (n = 24)	China	The experiences and perceptions of nursing were mostly negative, especially regarding recruitment programs, gender bias in nursing education, and societal views on nursing work.
**Rajapaksa & Rothstein (2009) (20)**	Survey	Male and female nurses (n = 1589)	The USA	Men were more likely than women to cite better pays as a reason for leaving the nursing profession.
**Varaei et al. (2012) (19)**	Survey	Baccalaureate nurses (n = 220)	Iran	Nursing was perceived as a suitable profession for both men and women
**Curtis et al. (2009) (7)**	Survey	Male and female qualified nurses in four censuses	The UK	During the 10-year period, the working life of a male nurse decreased by 9 years compared to expected working life of a female nurse which decreased by only one year.
**McLaughlin et al. (2010) (8)**	Longitudinal study	Male and female nurses (n = 384)	The UK	The findings indicated that males were more likely to leave the program than females.
**Jalali et al. (2011) (22)**	Mixed method	Male and female nurses (n = 97)	Iran	Findings revealed some themes, as, responsibility for society, responsibility for colleagues, necessity of attendance of men in nursing profession, and constraints in the implementation of services.
**Yang et al. (2004) (21)**	Descriptive qualitative	Male nurses (n = 15)	Taiwan	Male nurses utilized various strategies improving their knowledge and skills to attain higher levels of satisfaction and enhanced opportunities for promotion such as choosing specialty areas.
**Fooladi (2003) (15)**	Ethnography	Nursing faculty members and students (n = 11)	Iran	The author identified gender differences in care and compassion, spirituality, monetary motives, and practice preference.
**Muldoon & Reilly (2003) (25)**	Survey	Male nurses (n = 384)	The UK	Gender role orientation had a greater impact on students’ career preferences than gender by itself.
**Miers et al. (2007) (24)**	Content analysis	Male and female students in health care professions (n = 775)	The UK	Altruism was the most frequently cited reason for desire to join a non-medical health profession.
**Meadus & Twomey (2007) (10)**	Survey	Male nurses (n = 250)	Canada	Job security, career opportunities, and payment were the most common reasons for entering nursing.
**Zysberg & Berry (2005) (28)**	Survey	Male and female nursing students (n = 160)	The USA	Men put greater emphasis on aspects such as salary, job security, and social image of the profession.
**Whittock & Leonard (2003) (26)**	Mixed method	Male nurses (n = 60)	The UK	Lack of career advice for young men in relation to the nursing profession reported by authors.
**Boughn (2001) (23)**	Grounded theory	Male and female nurses (n = 28)	The USA	Findings showed that there was a strong contrast between male and female students regarding practical motivations for choosing nursing.
**Mullan & Harrison (2008) (2)**	Survey	Male and female nurses (n = 273)	Australia	The results of this study indicated that it is unlikely to be the individual differences between males and females that determine their career progress.
**Zamanzadeh et al. (2013) (27)**	Content analysis	Male nurses (n = 18)	Iran	Practical motivations such as job security were important factors in choosing nursing.
**Chou and Lee (2007) (29)**	Content analysis	Male nursing students (n = 10)	Taiwan	The results were classified into three major categories: professional ambition choice, professional gender expectation, and development of nursing philosophy following a primary clinical internship.
**McMillian et al. (2006) (32)**	Survey	Female nurses (n = 105)	The USA	The duration in which female nurses work with a male nurse, explains significant variance in acceptance score.
**Vaismoradi et al. (2011) (16)**	Content analysis	Nursing students (n = 14)	Iran	The main barriers for the development of professional identity was related to lack of clear and acceptable public image of nursing
**Grady et al. (2008) (30)**	Phenomenology	Nursing faculty members (n = 6)	The USA	Findings revealed some themes, as, altruism, attainment agency, ambiguity, and anecdotes.
**Jinks & Bradley (2004) (31)**	Survey	Male and female nursing students (n = 96)	The UK	Findings revealed significant differences compared to a similar study performed in 1992 regarding gender and nursing stereotypes.
**Miller (2004) (37)**	Grounded theory	Male nurses (n = 7)	The USA	Masculine infusion theory was emerged from data.
**Patterson & Morin (2002) (34)**	Phenomenology	Male nurses (n = 8)	The USA	Participants expressed concerns about meeting clinical objectives and personal goals due to their gender.
**Fisher (2009) (3)**	Theorized life history method	Male nurses (n = 21)	The UK	Male nurses’ clinical practice is both shaped and constrained by gender negotiations.
**Evans (2002) (12)**	Thematic analysis	Male nurses (n = 8)	Canada	Participants cited some stereotypes that make intricate and contradictory situations of acceptance, rejection and suspicion of men as nurturers and caregivers.
**Milligan (2001) (33)**	Phenomenology	Male nurses (n = 8)	The UK	Participants reported feelings of isolation and conflict when providing personal care, especially to female patients.
**Simpson (2005) (35)**	Content analysis	Nurses, cabin crew, librarians and primary school teachers (n = 40)	The UK	Role strain was a prevailing experience for men in nontraditional careers.
**Smith (2006) (36)**	Mixed method	Male nursing students (n = 29)	The USA	Participants mentioned difficulty in balancing school, family, and work as their main concerns.
**Evans & Frank (2003) (14)**	Descriptive qualitative	Male nurses (n = 8)	Canada	The findings revealed contradictions and tensions of men’s lives in non-traditional occupations.

## 3. Results

### 3.1. Characteristic of Men Who Enter Nursing

There is no well-documented literature about demographical data of typical men who enter the nursing profession. Most of the descriptive qualitative researches addressed the motivations of men entering nursing (23, 24, 27). Some quantitative researches are also available, which describe the typical characteristics of males who enter nursing (2, 10, 28). Men who enter nursing were mostly married and older than their female colleagues (2, 28), predominantly low-to middle class, and most came to nursing from another career (37). However, in most eastern countries including Iran, students are required to participate in a national entrance exam, to enroll at a university (18, 21, 27). It has been reported that most male candidates feel disappointed once they find out that nursing is their major (15, 18, 27). In a study conducted by Wang et al. in Chinese patriarchal society, of 15 male nursing students, only one participant chose nursing as his major, and the others chose clinical medicine or other majors of the university and were assigned to the nursing major (18). In an Iranian qualitative study, most of the participants were from middle and middle-to low socio-economic backgrounds, and most of them generally made uninformed choices about their career (27). Desire to avoid the military draft was also reported as a reason to accept any courses including nursing by male high school students in some eastern countries (21, 27). Regarding personality characteristics of men who enter nursing professions, Stott suggested that those men choosing and remaining in nursing could be more “androgynous” in orientation than men in other typically male-dominated professions; hence, suffering less role strain when performing their occupational roles (13). There are also some researches exploring men experiences in other female-dominated professions. The results of Simpson's study who explored male workers in four occupational groups including nurses, flight attendant, librarian, and elementary school teachers, suggested three typologies of men in female predominance occupations: seekers (who actively look for the career), finders (who find the job in the process of making general career decisions), and settlers (who stay into the non-traditional career after a period of time in mainly male dominated occupations). Simpson concluded that despite the comfort men feel in feminine professions, they adopt a variety of strategies such as status enhancement and distancing from the female colleagues, to re-establish a masculinity that has been undermined by the 'feminine' nature of their work (35).

### 3.2. Motivation Influences Men to Enter Nursing

Motivation is the internal and external drives that influence men's decision to enter nursing. Some quantitative researches exist regarding the reasons why men enter nursing (10, 25, 28). Most of our knowledge about motivation resides in qualitative studies (23, 24, 26, 27). Influencing factors on men’s contribution in nursing can be practical, as well as personal. Practical factors are associated with a decision which most likely has a favorable outcome for the person, such as salary, working conditions and job security (23). Personal factors can be considered as those reasons which fulfill some internal drives or needs such as, altruistic desires (24). The three main reasons discovered throughout the literature on men entering nursing are: significant others, personal, and practical motivations.

#### 3.2.1. Significant Others 

Parents, families and friends play a significant role in the occupational aspirations and career choices of their children (26). Chou and Lee found that approval of family and friends had a generally positive influence on men’s decision to enter nursing (29). However, most of the participants in Zamanzadeh et al. study stated that they had not thought of being a nurse before entering university, some of them told stories about high school counselors and others such as their friends who were nurses, and parents, playing a crucial role in their career decisions (27). Men were also faced with a negative feedback from other males, including fathers and male friends, when sharing their decision to enter nursing (14). Most male nurses in O’Lynn study, knew a male nurse prior to enrolling, and acknowledged that peer support from other male nurses were important in their educational experience (11). What can be inferred from the literature is that support from significant others close to the male nurse is an influential factor when choosing nursing as a profession. Nevertheless, most of this support comes from females who are close to men that are interested in pursuing a nursing profession (11, 13, 14). In general, females tend to accept working in non-traditional occupations more than men (32).

#### 3.2.2. Personal and Practical Motivations

Boughn reported that when participants were asked to rate their reasons for entering nursing, females rated individual fulfillment higher than males, and men were more likely to rate career opportunities and salary as more important motivators for entrance (23). Despite the fact that most quantitative studies, mentioned practical reasons as motivation behind men entering nursing (10, 20, 28), altruistic desires were one of the major influencing factors for men who enter nursing in qualitative studies (23, 26). These studies revealed that men nurses usually state the desire to help others. Boughn reported that regardless of having an altruistic desire by female nurses, they are more likely to be younger, and seek to achieve a sense of empowerment from entering nursing (23). Zamanzadeh et al., who studied the reasons for choosing nursing as a career by Iranian male nurses, found that in most cases the desire to care for others was not the primary reason for choosing nursing (27). This is supported by Fooladi who reported that Iranian male nursing students lack interest in compassionate nursing care and view nursing as a source of income and security (15). In general, male and female nurses have some different aspirations for entering nursing. For men, practical motivation such as, job security, and diversity nursing offers are of utmost importance, and women are attracted to nursing mostly for altruistic desires and feelings of self-empowerment (23, 28).

### 3.3. Challenges for Men Entering into Nursing

Most studies analyzed for this review revealed many challenges faced by men entering or working in a profession in which women are predominant (1, 3, 11, 14, 17, 30, 32, 36). However, most of these challenges are experienced by male nurses during their educational programs (11, 13, 17). These educational barriers that are described by male nurses in O’Lynn study included several aspects such as “no mentorship program” for male students, “no male faculty”, “inadequate opportunity” to work with men nurses in the clinical areas, and no guidance on “the use of touch” (11). Main components of difficulties and challenges specific to male nurses in the literature include: role strain, nursing as a feminine profession, and caring.

#### 3.3.1. Role Strain

Role strain was defined by Stott as “when an individual is likely to experience tension when coping with the requirements of incompatible roles” (13). Among male nursing students, role strain takes a variety of forms. Smith reported that role strain is usually experienced by men students as difficulty of balancing school, family, and work (36). For male nurses, role strain can originate from a range of sources, such as masculine stereotypes (12, 14), male nurses’ contribution to heavy manual work (3), and proper use of touch (13). Despite that male nursing students may feel considerable role strains, it has been shown to reduce as they step forward in their educational programs (1). Stott suggested that suffering less role strain by some male nursing students may be due to their “androgynous” orientation (13). In addition, male nurses gravitate toward technical, nonclinical, or high acuity areas to cope with role strain in a female dominated profession (14). Miller also reported that male nurses were drawn towards management and specialized areas, not only due to economic reasons, but also to reduce role strain (37). Findings of some reviewed researches cited male preference of working in technical areas such as, emergency, ICU, operating room, and anesthetics (3, 21, 25, 33). Iranian male nurses were gravitated toward positions suitable to their masculine nature in management, emergency and intensive care areas to gain physician’s trust and community’s respect (5, 15, 27). Evans stated that the male tendency to such technical areas may be due to the fact that such roles appears more congruent with the masculine role. This helps men reduce the role conflict they may experience, and disperses any stereotypical labels (12). Working in such areas seems to decrease the extent of role strain, because not only less personal care is needed, but also nurses are less required to be dressed in traditional nurse uniforms (13). Gender roles are the responsibilities, attitudes, and typical behaviors associated with a person’s gender, for example, men are physicians and women are nurses. The man is the provider of a family, and the woman acts as a caregiver (35). It appears that males experience role strain from both changing roles within the family and also conflicts which come up from being in a female dominated profession (13).

#### 3.3.2. Nursing as a Feminine Profession 

One of the major obstacles that may dissuade men to enter the nursing profession is the traditional female image (14). The feminine nature of nursing has been so prevailing, that the caring image of the profession has been used to symbolize the epitome of femininity (11). It seems that the social construction of nursing as a female profession creates difficulties for male nurses and their caring abilities (14). Within the qualitative literature, men often report their needs in educational and clinical settings as being ignored (12, 33, 34). Male nursing students’ experience of isolation may come from being male in a predominantly female career, and they express a desire to interact more often with male role models (10, 13). Male nurses felt more isolation and challenges than their female counterparts, especially when providing care to females (2, 3, 14, 33). However, Varaei et al. in a quantitative study reported that most nurses perceived nursing as a suitable career for both Iranian men and women (19); the findings of related qualitative studies in Iran were somewhat different. Iranian male nurses in Zamanzadeh et al. study reported that one of the most important factors hindering men from choosing the nursing profession is the public's image of nursing as a female occupation, and nurses being portrayed as physician subordinates (27). Accordingly, male nursing students in Vaismoradi et al. study described friends’ or relatives’ negative reactions to their choice of nursing (16). Despite men nurses altruism and caring motivation cited in literature for choosing nursing (23, 26). The physical nature of men’s bodies, size, muscle mass, and strength cause some hinders for male nurses especially when providing care for female patients (3). The physical nature of men leads to an informal division of labor where men are expected to perform heavy physical work and in providing safety for their female colleagues (3, 4, 14). 

#### 3.3.3. Male Nurses Caring Abilities

Although caring is the essence and one of the most basic nursing values, men's ability to care is questioned in some nursing literature (3, 14, 33). As caring is traditionally recognized as a feminine quality, some researcher explored developing caring skills in male nurses (13, 30, 33). Regardless of the general perception that women are more caring than men (4, 12), in Zamanzadeh et al. Whittock and Leonard qualitative studies, male nurses did not perceive caring as an inherently feminine trait and suggested that male nurses can be as caring as females (26, 27). However, male nursing students expressed concerns related to role strain and stereotyping, particularly regarding caring behaviors (12). Male students stated that patients expected to be cared for by female nurses in the clinical setting, and this is the factor that caused them to always adjust regarding their interaction with patients and also other females in clinical experience (13). In Milligan study who explored the concept of care among male nurses, participants addressed factors seen as integral to care including: the perceived expectation that they should deal with emotional work and do not show emotions in public; struggle with the more bodily aspects of work, such as manual handling and caring confused/aggressive patients; issues related to their gender, or social perceptions of their gender; and an association with technical tasks (33). One of the other challenges faced by male nurses during performing caring roles is touching (12). As nurses are often required to come into intimate contact with patients, literature noticed various aspects of this issue (3, 13, 33). According to Evans, male nurse have reported that they are discouraged from going into high-touch fields, and even male nurses with five or more years of clinical experience still felt difficulty with caring procedures needing intimate contact, and this issue gravitates them to low-touch fields such as management areas (14). Regarding to the fact that most caring role are taught to male students by female mentors, and most nursing faculties fail to consider the different ways male and female students learn and express caring, the literature suggested that male students need more male role-models to assist them in adjusting with this demand of their roles (12, 13, 30). 

### 3.4. Opportunities for Men Within Nursing

Despite the Barriers, males continue to enter nursing and also advise others to choose nursing, which implies the advantages men achieve when entering nursing (10). As mentioned before, male are facing unique challenges related to their gender. The paradox of this situation is the advantages of being male in a female-dominated profession (14, 15, 22). Men who enter the nursing profession tend to have faster and more straightforward career progression than women (25). Fooladi explored Iranian nursing education and practice settings by using an ethnographical method, and indicated advantages male nurses are attaining in the Iranian patriarchal health system such as, assuming leadership positions (15). Men also found that they may be better treated by physicians, felt as more stable occupation and believed that their career paths are more bounteous with better opportunities of progression compared with some other male dominant professions (17, 21). Furthermore, men typically found that practicing in certain areas such as mental health, critical care, anesthesia, emergency care, and administration can provide better pay, or they are not required to touch frequently (13). Men seem to fall into these various technical and non-technical areas from the influence of their female colleagues and nursing instructors and managers. Male nurses move towards areas such as the operating room and emergency care may be due to support from male physicians (14, 17). Men were also found to have a more positive attitude towards their ability to succeed when they are working in such technical areas (31). Stott reported that male students felt they were less likely to receive disciplinary action from their female nursing instructors when mistakes were made on the clinical setting (13). According to Evans and Frank, the quantity and quality of work expected from male and female nurses were not the same. Many of the participants reported that in many instances they were not expected to do as much “feminine” works and often, female nurses would intervene and perform those works instead of them (14).

## 4. Conclusions

Most reviewed literature identified the unique difficulties and challenges experienced by men in educational and clinical settings. Men are marginalized, and sometimes not accepted, within nursing. Also, male nurses must confront challenging traditional gender-defined roles and stereotypes from the larger society when choosing to enter a female-dominated profession. This requires special considerations, of which using media for truthfully introducing the profession to the public can be helpful. Due to male nurses’ impacts on career advancement and also the importance of gendered nursing practice that allows male nurses to care for male patients and female nurses to attend to female patients, males’ recruitment and retention programs must be followed hastily. Teaching caring roles to male nursing students should be provided by male mentor and role models in a scientific and rational manner. Differences in learning and expressions of male and female caring should be appreciated by female instructors, as well male students could not be marginalized by being expected to adhere to feminine expressions of caring. However, it is necessary to take into account applicants’ abilities and personality characteristics. Regarding the nature of nurses work and role strain reported in the literature, informing high school students before applying for university courses, would prevent waste of training costs due to male nurses’ attrition rate early in their career life, as well as creating an environment to persuade those males who have some extent of personal interest to enter and pursue nursing. However, male entrance into nursing profession still has some advantages despite barriers they are faced with. Job security is one of those factors which have a major impact on choosing nursing by male nurses. However, entering nursing based on influence of more controlled motives such as, job security or advice from family and friends is unlikely to be beneficial to the nursing profession in the long term. Finally, most of the reviewed articles investigating the experiences of male nurses in educational and practice settings were performed in western countries mainly the USA, Canada, and the UK. In addition, cultural differences and methodological limitations of these studies make it difficult to generalize those findings for other countries. Studies conducted in Iran and other eastern countries were exclusively performed using quantitative methodology and small samples. In relation to future research, exploring gender-based experiences and motivators for entering nursing by Iranian male nurses’ using qualitative methodology are required. In addition, replicating these studies in female nurses and comparing the findings can help better understand the role of gender in nursing.
